# Supplementary material for: Cooperative supramolecular polymerization of styrylpyrenes for color-dependent circularly polarized luminescence and photocycloaddition
Source: Nat Commun. 2023 Dec 4;14:8022. doi: 10.1038/s41467-023-43830-x (PMC10696047; doi:10.1038/s41467-023-43830-x)
Supplement: Supplementary file 1 — Supplementary Information [file 41467_2023_43830_MOESM1_ESM.pdf]

Supplementary Information

Cooperative Supramolecular Polymerization of Styrylpyrenes for Color-Dependent Circularly Polarized Luminescence and Photocycloaddition

Yuan et al.

Supplementary Figures and Tables

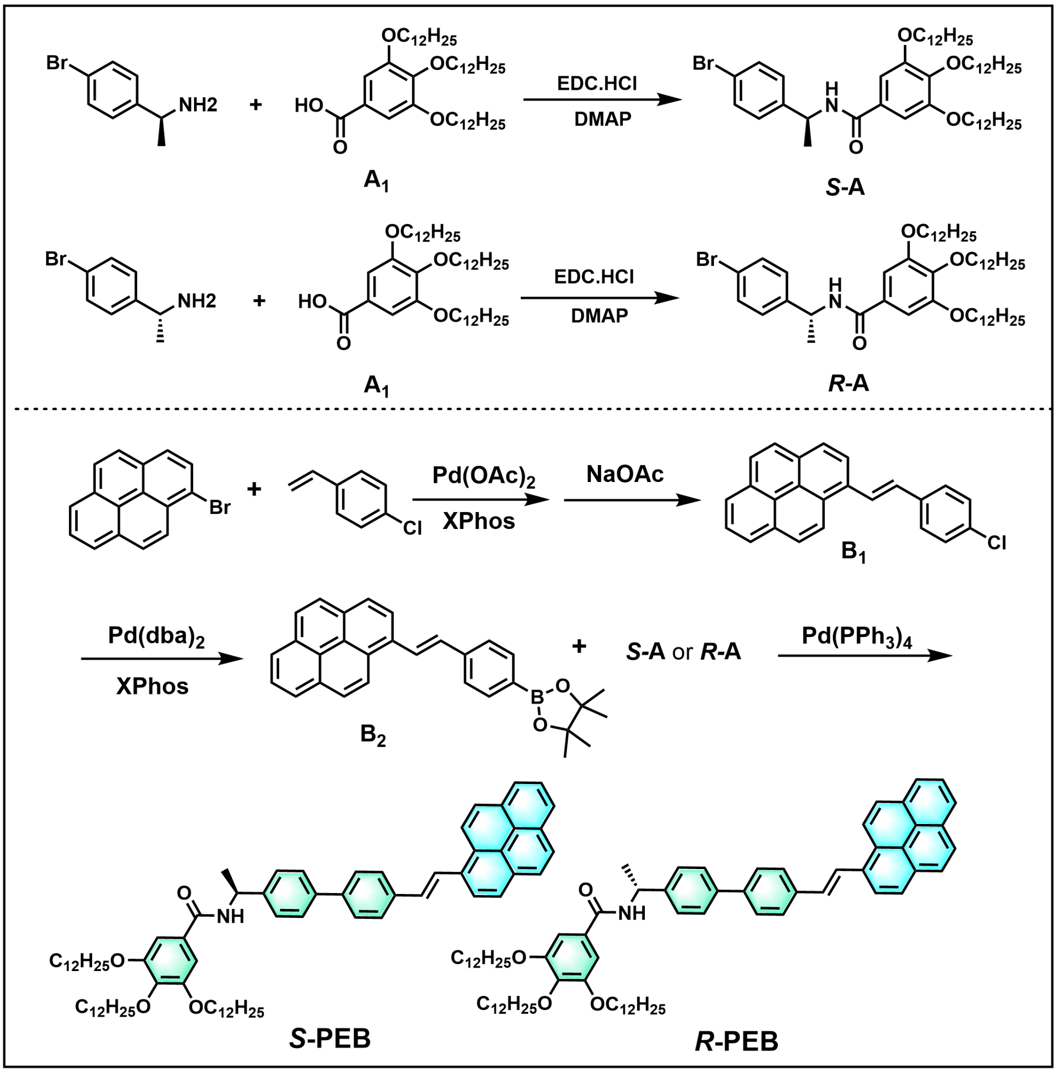

Supplementary Figure 1. Synthetic routes of *S*-PEB and *R*-PEB.

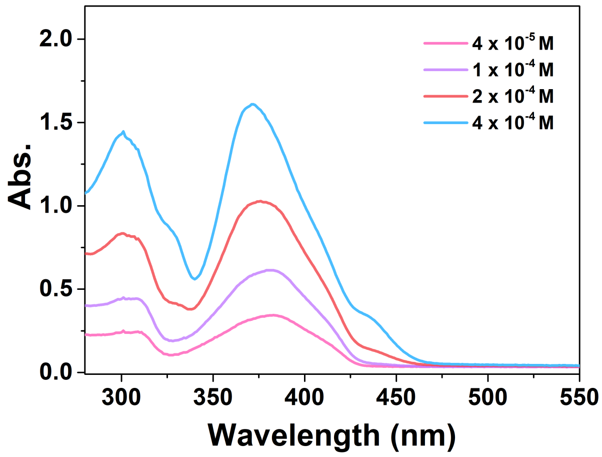

Supplementary Figure 2. Varied-concentration UV-vis spectra of *S*-PEB in MCH/TCE (24:1, v/v) solution.

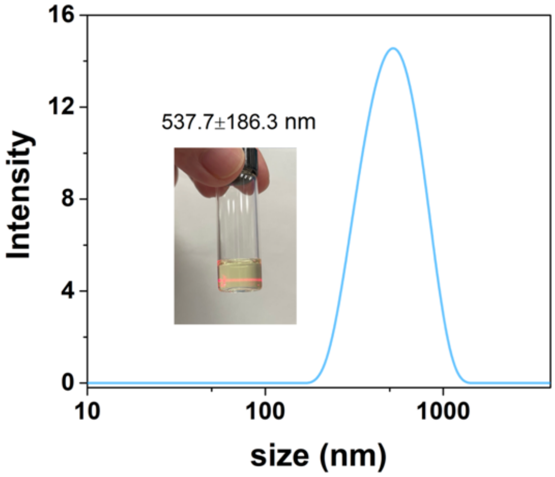

Supplementary Figure 3. Dynamic light scattering (DLS) size distribution of *S*-PEB in MCH/TCE (24:1, v/v) solution (C = 4 × 10^{-4} M). The average size of aggregates is 537.7 ± 186.3 nm. The inset is the photograph of a Tyndall effect for *S*-PEB in MCH/TCE solution.

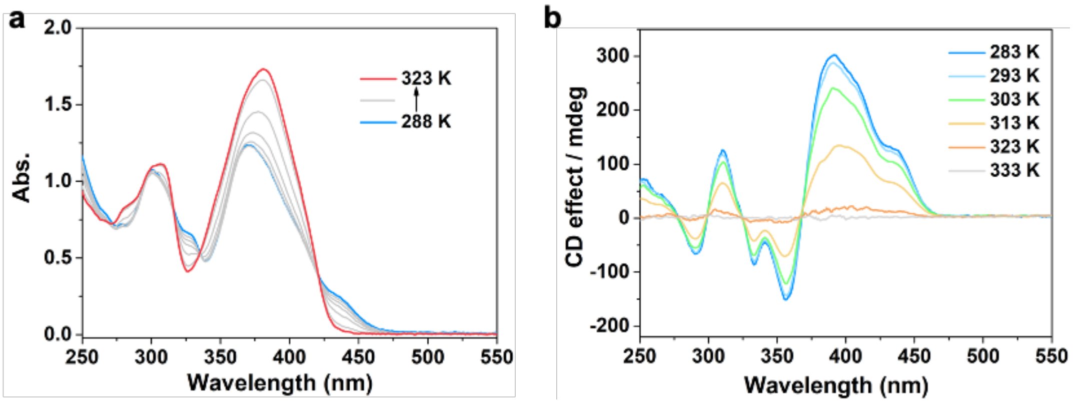

Supplementary Figure 4. **Varied-temperature UV-vis absorption.** (a) Varied-temperature UV-vis absorption of *S*-PEB upon heating from 288 K to 323 K in MCH/TCE (24:1, v/v) solution. (b) Varied-temperature CD spectra of *S*-PEB upon heating from 283 K to 333 K in MCH/TCE (24:1, v/v) solution. All the concentrations are 4 × 10^{-4} M.

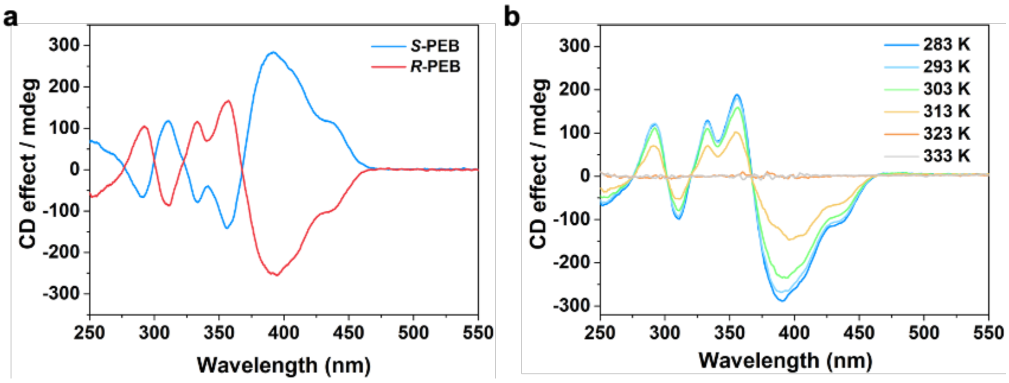

Supplementary Figure 5. **CD spectra.** (a) CD spectra of *R*-PEB and *S*-PEB in MCH/TCE (24:1, v/v) solution at 298 K. (b) Varied-temperature CD spectra of *R*-PEB upon heating from 283 K to 333 K in MCH/TCE (24:1, v/v) solution. All the concentrations are 4 × 10^{-4} M.

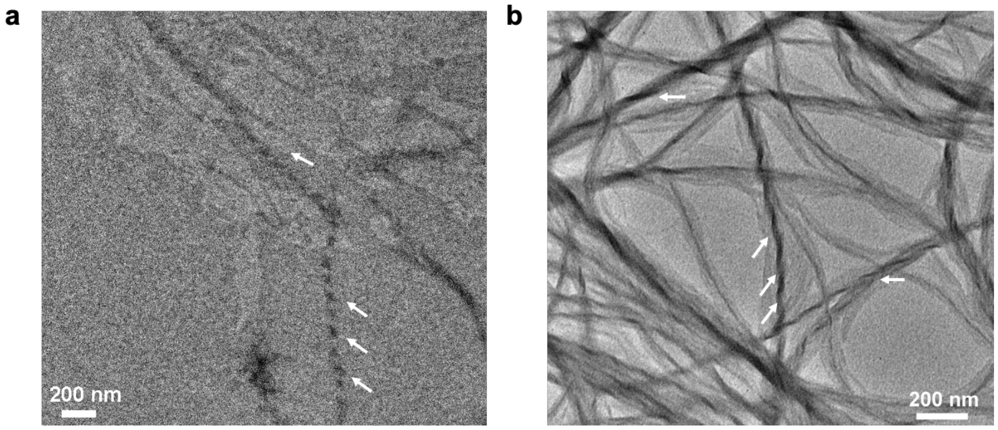

Supplementary Figure 6. **TEM images.** Supramolecular polymers (a) *S*-PEB and (b) *R*-PEB prepared from MCH/TCE (24:1, v/v) solution ($C = 4 \times 10^{-4}$ M).

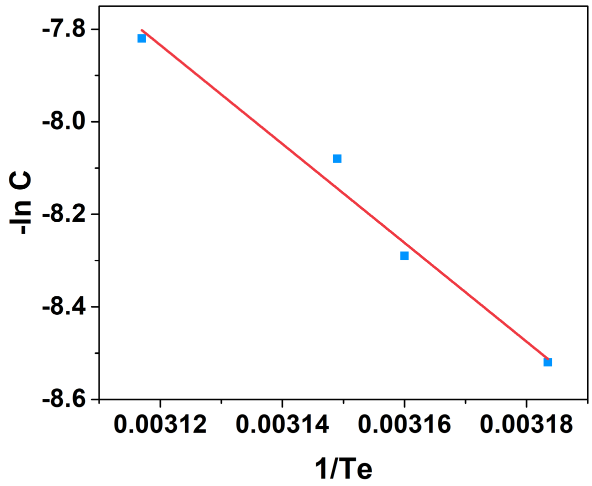

Supplementary Figure 7. Van't Hoff plot for the supramolecular self-assembled process of *S*-PEB. The red color line denotes the linear fitting curve.

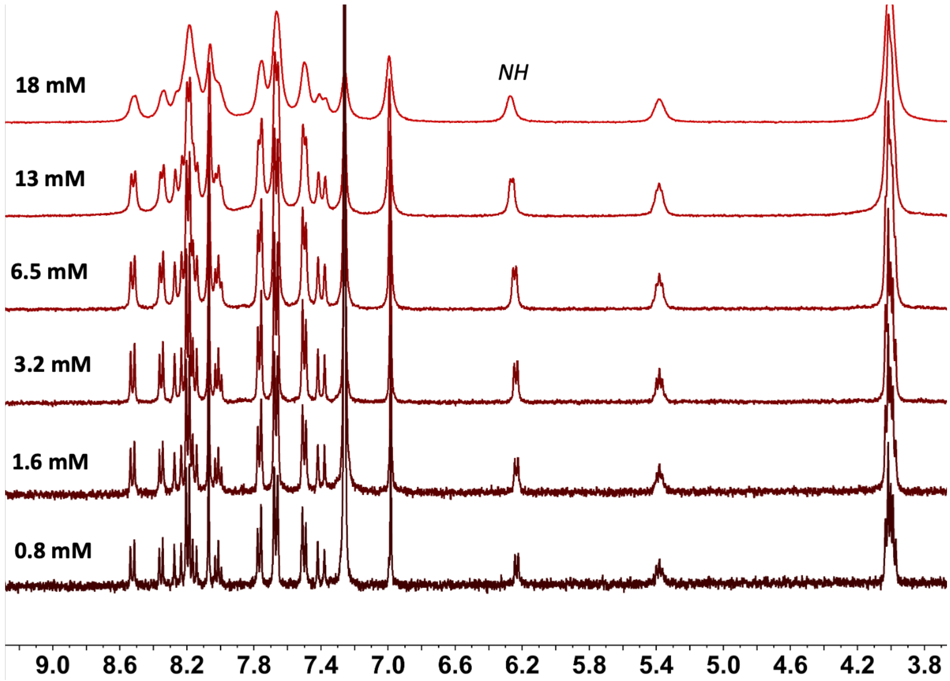

Supplementary Figure 8. Concentration-dependent ^{1}H NMR spectra of *S*-PEB upon varying the concentration from 0.8 mM to 18 mM in $CDCl_3$. It was observed that the resonance signals for NH protons and aromatic moiety protons were almost unchanged upon increasing the concentration. On this base, it is reasonable that neither intermolecular hydrogen bonding nor π-π interaction is the main interaction for the supramolecular polymerization process.[1]

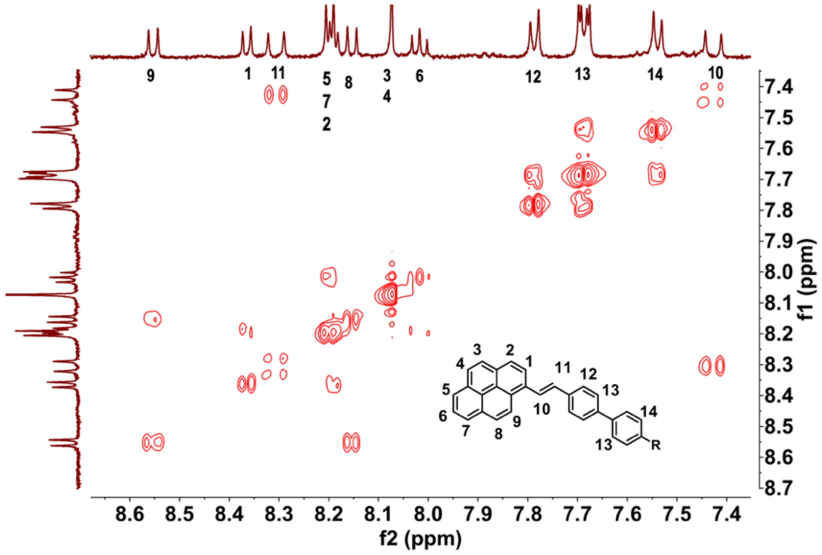

Supplementary Figure 9. Enlarged view of the 2D COSY measurement of *S*-PEB in cyclohexane-d_{12}/$CDCl_3$ (v/v = 2:1, C = 5 × 10^{-3} M).

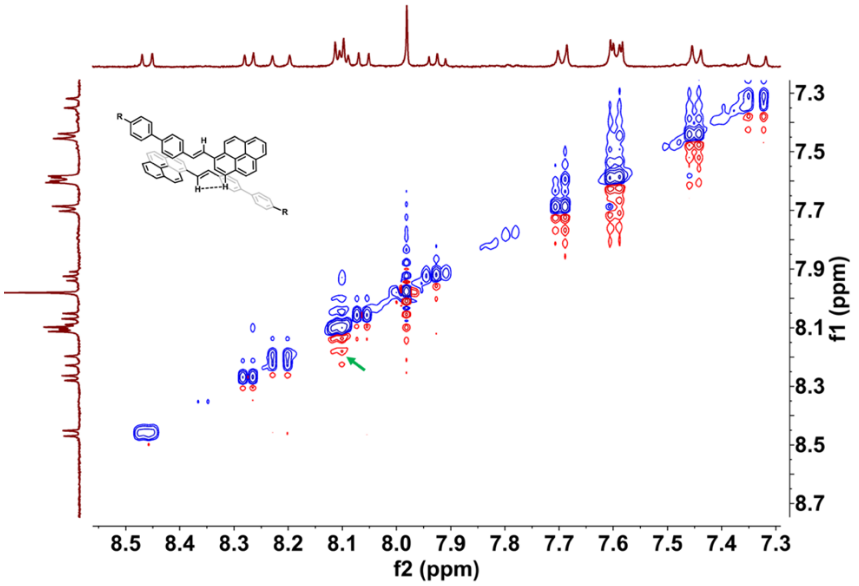

Supplementary Figure 10. Enlarged view of the 2D NOESY measurement of *S*-PEB in cyclohexane-d12/CDCl3 (v/v = 2:1, C = 5 × 10^{-3} M). The green arrow indicated that the interaction between the proton 2′ and 11′, suggesting the "twist-antiparallel" stacking pattern.

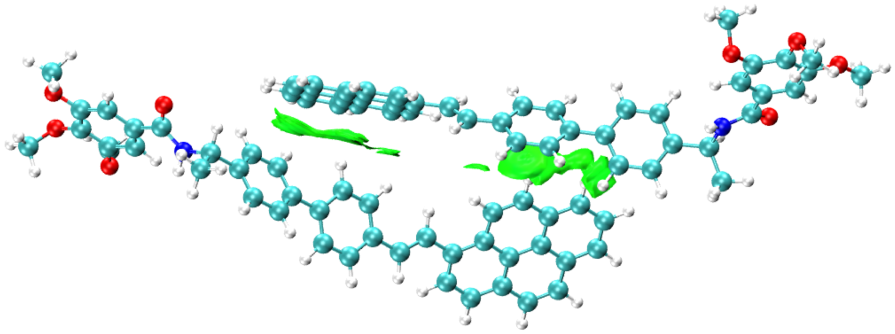

Supplementary Figure 11. Visualization of the isosurface for weak CH-π interactions by Multiwfn and VMD. The colormap for the isosurface has the same scheme as the scatter map for the top view. The green areas in the isosurface plots indicate weak CH-π interactions.[2]

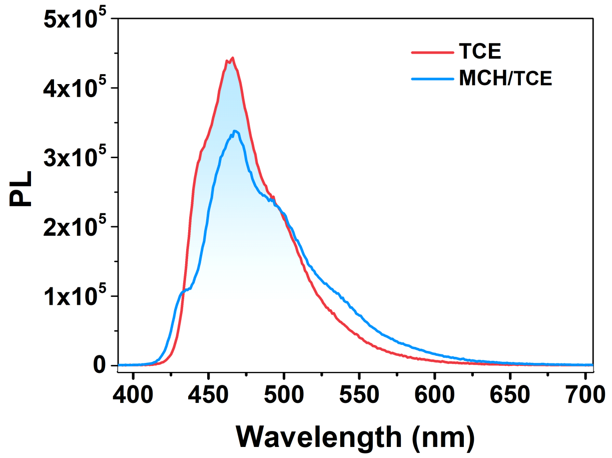

Supplementary Figure 12. Fluorescence spectra of *R*-PEB in dilute TCE and MCH/TCE (24:1, v/v) solution ($C = 4 \times 10^{-4}$ M).

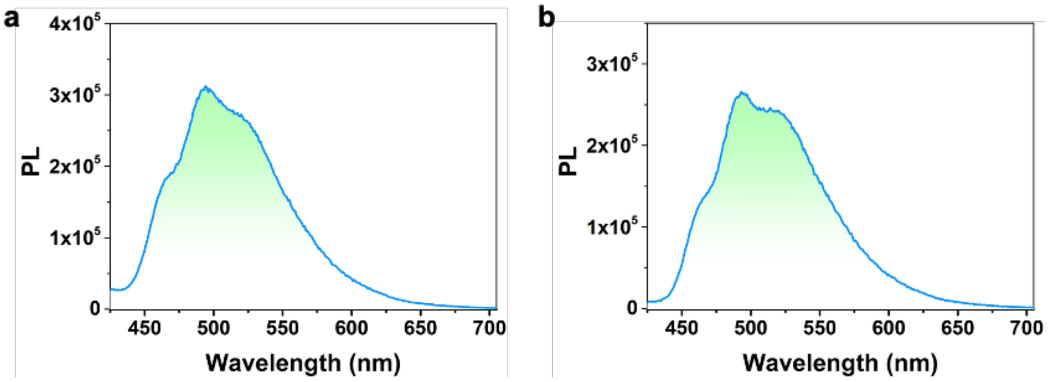

Supplementary Figure 13. **Fluorescence spectra.** Supramolecular gels (a) *S*-PEB and (b) *R*-PEB formed in MCH solution (11 mg/mL).

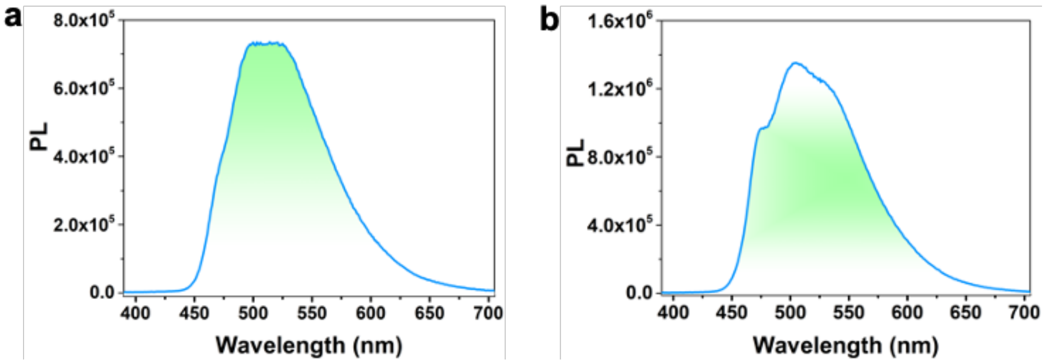

Supplementary Figure 14. **Fluorescence spectra.** Fluorescence spectra of (a) *S*-PEB and (b) *R*-PEB drop-casting films prepared from the MCH/TCE solution (24:1, v/v).

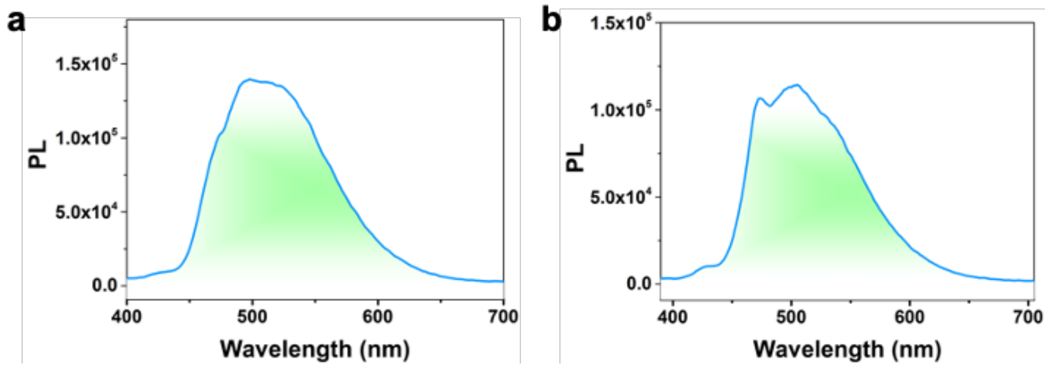

Supplementary Figure 15. **Fluorescence spectra.** Fluorescence spectra of (a) *S*-PEB@PMMA and (b) *R*-PEB@PMMA bulk films.

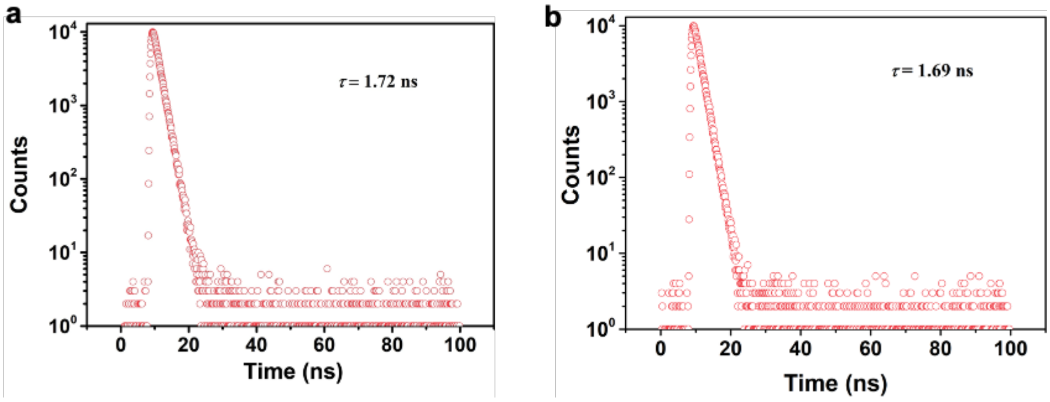

Supplementary Figure 16. **Fluorescence decay curves.** Fluorescence decay curves of (a) *S*-PEB and (b) *R*-PEB in TCE solution.

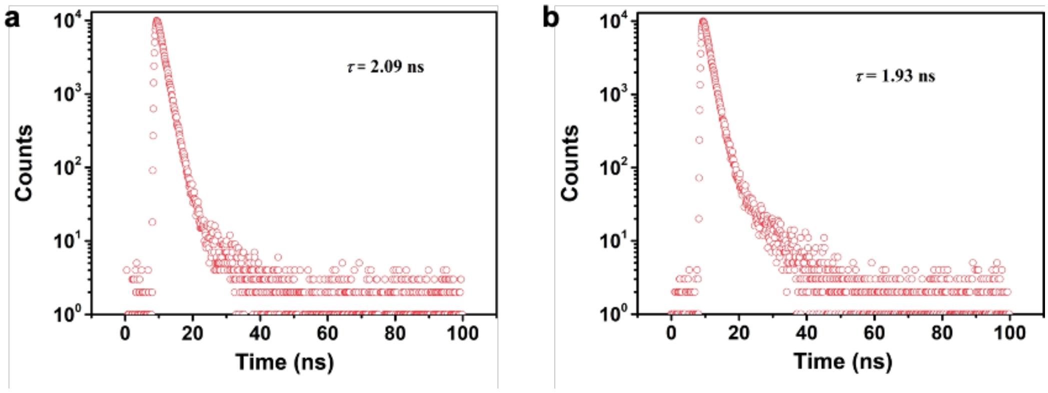

Supplementary Figure 17. **Fluorescence decay curves.** Fluorescence decay curves of (a) *S*-PEB and (b) *R*-PEB in MCH/TCE (24:1, v/v) solution.

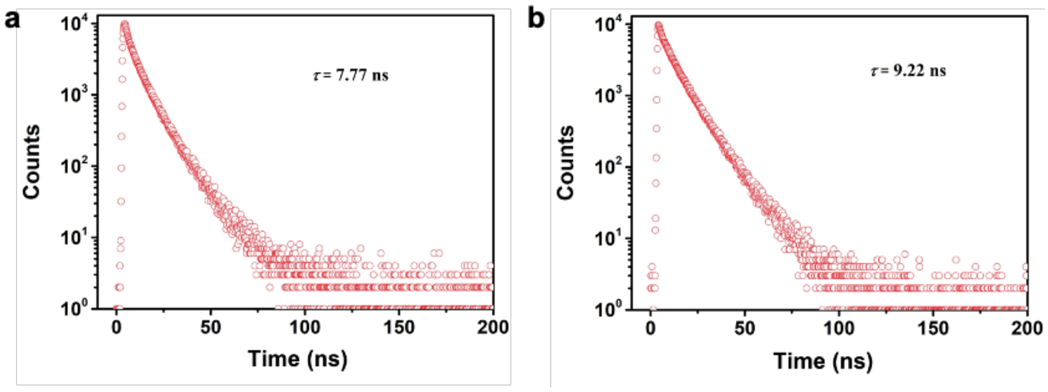

Supplementary Figure 18. **Fluorescence decay curves.** Fluorescence decay curves of (a) *S*-PEB and (b) *R*-PEB supramolecular gels formed in MCH solution (11 mg/mL).

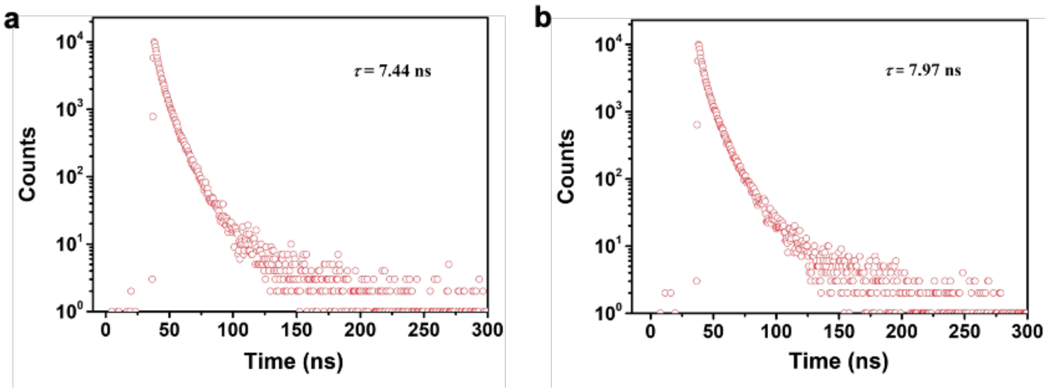

Supplementary Figure 19. **Fluorescence decay curves.** Fluorescence decay curves of (a) *S*-PEB and (b) *R*-PEB drop-casting films prepared from the MCH/TCE (24:1, v/v) solution.

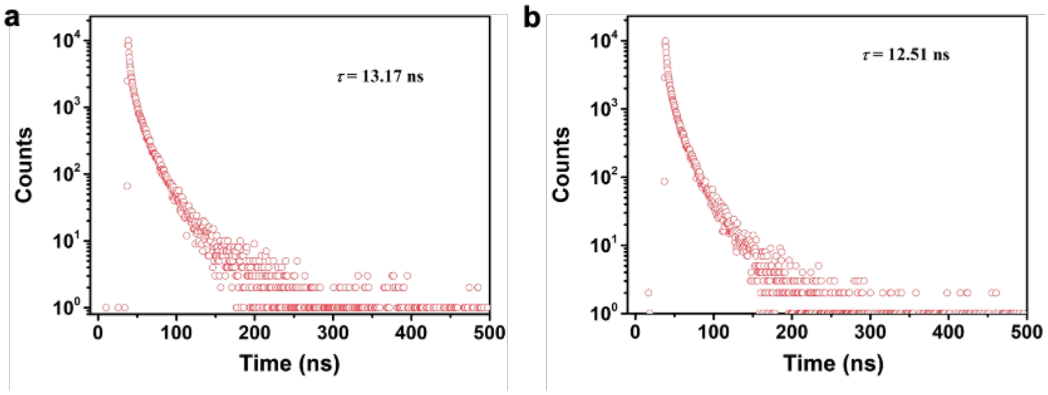

Supplementary Figure 20. **Fluorescence decay curves.** Fluorescence decay curves of (a) *S*-PEB@PMMA and (b) *R*-PEB@PMMA bulk films.

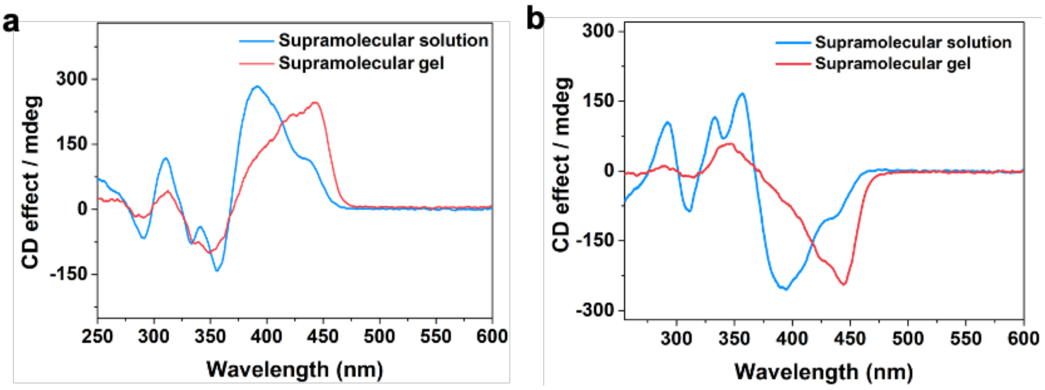

Supplementary Figure 21. **CD spectra.** CD spectra of (a) *S*-PEB and (b) *R*-PEB in the supramolecular solution and supramolecular gel states.

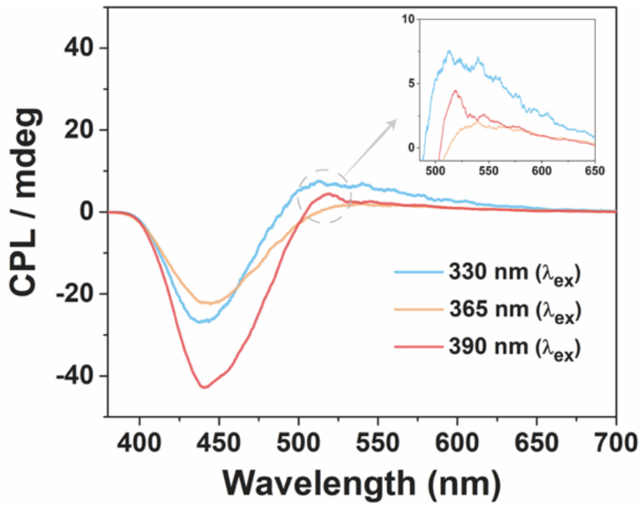

Supplementary Figure 22. CPL spectra of *S*-PEB in MCH/TCE (24:1, v/v) solution under various excitation wavelengths.

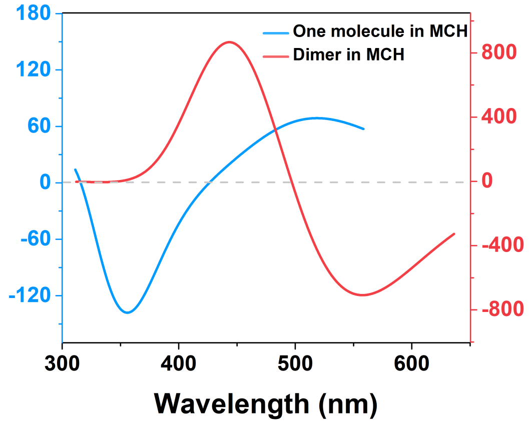

Supplementary Figure 23. Calculated CPL spectra of *S*-PEB in one molecule and dimer states. The calculations were performed at B3LYP/6-31g* level with solvent effect (methylcyclohexane as solvent).

Supplementary Table 1. Photophysical properties of *S*-PEB and *R*-PEB in different states.

Sample	PL Quantum Yield (Φ_Y) [a]	Lifetime of PL (ns)
S-PEB in TCE solution	70%	1.72
R-PEB in TCE solution	73%	1.69
S-PEB in MCH/TCE solution	69%	2.09
R-PEB in MCH/TCE solution	68%	1.93
S-PEB gel	39%	7.77
R-PEB gel	38%	9.22
S-PEB drop-casting film	[b]	7.44
R-PEB drop-casting film	[b]	7.97
S-PEB@PMMA film	26%	13.17
R-PEB@PMMA film	28%	12.51

[a] Absolute fluorescence quantum yields (Φ_Y) were determined by an integrating sphere method. [b] Unable to accurately test due to few samples.

Supplementary Table 2. Summarized circularly polarized luminescence performance of *S*-PEB and *R*-PEB in different states.

Sample	λ_{max}	g_{lum}	Φ_Y
S-PEB supramolecular solution	441	-1.1×10^{-2}	69%
R-PEB supramolecular solution	442	1.1×10^{-2}	68%
S-PEB supramolecular gel	509	5.3×10^{-3}	39%
R-PEB supramolecular gel	508	-4.9×10^{-3}	38%
S-PEB drop-casting film	513	3.6×10^{-3}	[a]
R-PEB drop-casting film	513	-3.0×10^{-3}	[a]
S-PEB@PMMA film	510	1.3×10^{-3}	26%
R-PEB@PMMA film	503	-1.0×10^{-3}	28%

[a] Unable to accurately test due to few samples.

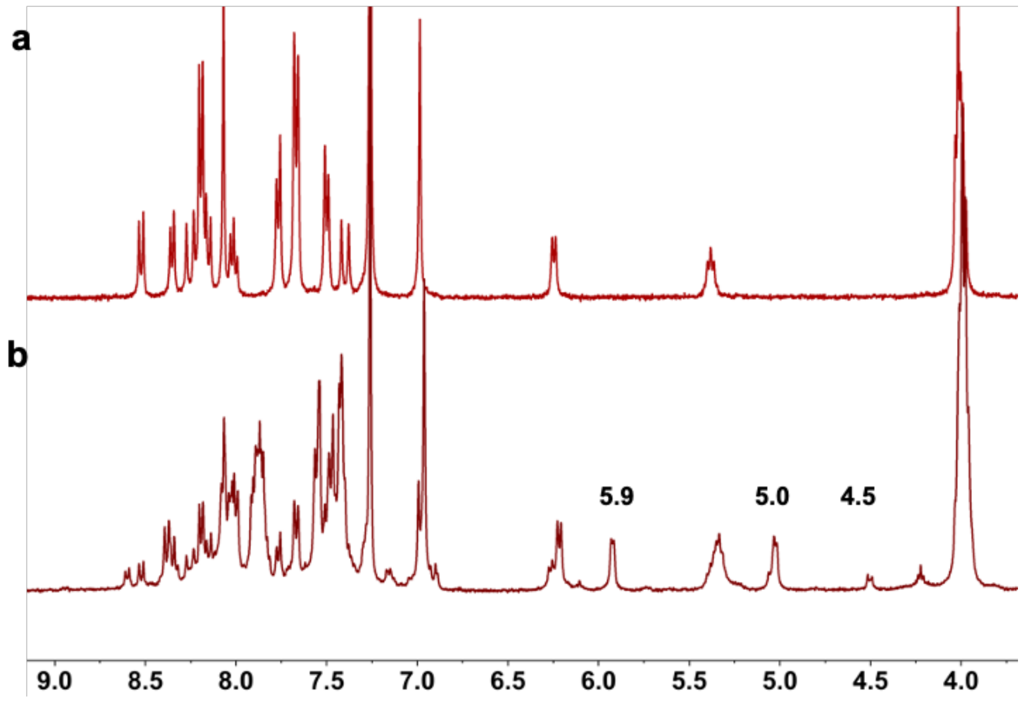

Supplementary Figure 24. **^{1}H NMR spectra.** (a) Partial ^{1}H NMR spectra of *S*-PEB in the absence of light irradiation, and (b) irradiating the MCH/TCE (24:1, v/v) solution of *S*-PEB for 55 min and evaporating the solvent, with the subsequent addition of $CDCl_3$ for NMR measurement. The new peaks at 4.5, 5.0, and 5.9 ppm suggested that [2+2] cycloaddition, rather than *cis*-*trans* isomerization, preferred to occur for *S*-PEB in the supramolecular solution upon irradiation ($C = 5 \times 10^{-4}$ M, V = 20 mL).

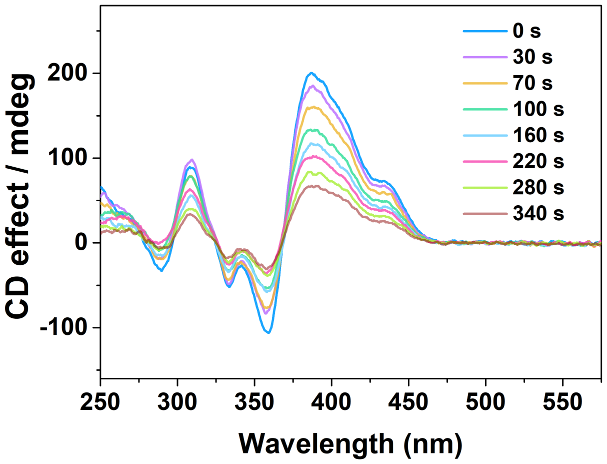

Supplementary Figure 25. CD spectral changes of *S*-PEB in MCH/TCE (24:1, v/v) solution upon 405 nm light source irradiation ($C = 4 \times 10^{-4}$ M, optical path length: 1 mm).

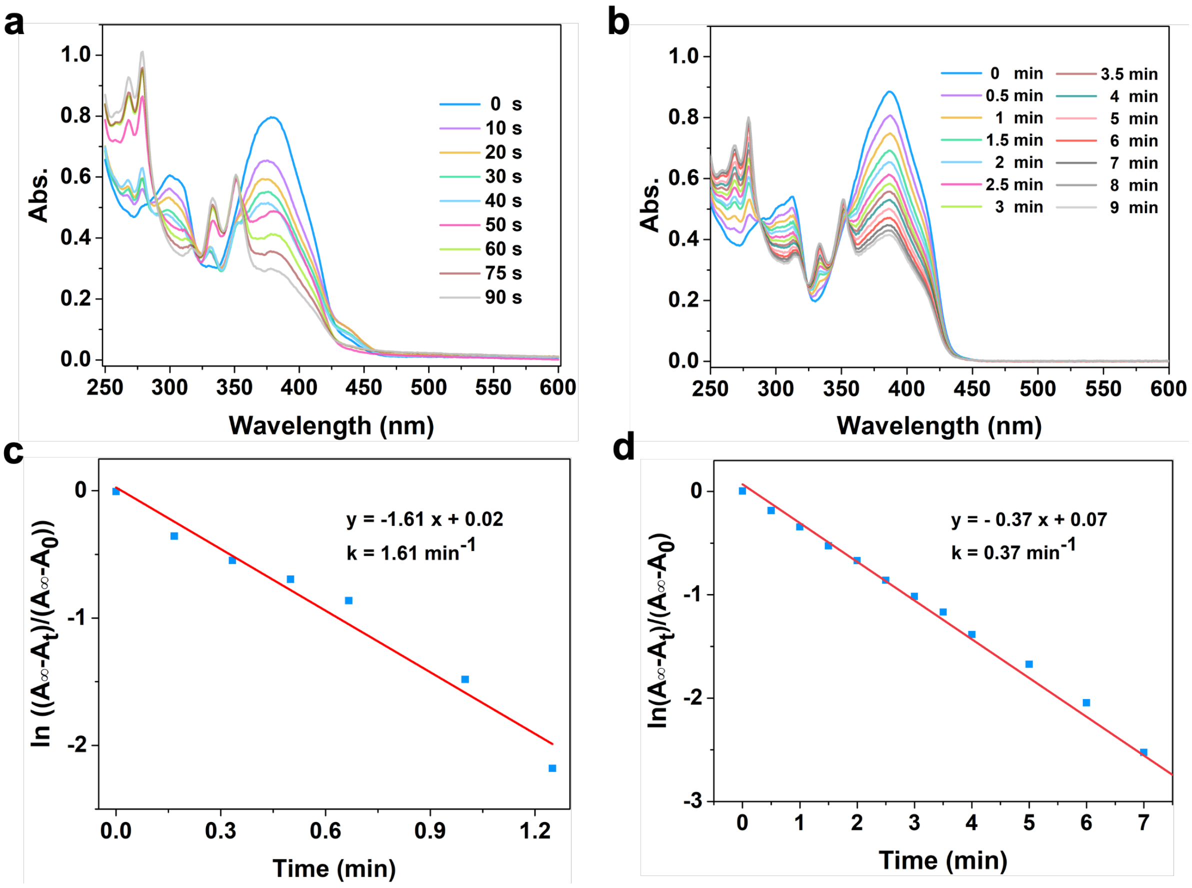

Supplementary Figure 26. **Time-dependent UV-vis spectra and kinetics.** Time-dependent UV-vis spectra of *S*-PEB in (a) MCH/THF (24:1, v/v) and (b) THF solution upon 405 nm light source irradiation. Kinetic studies for the [2+2] cycloaddition reaction of *S*-PEB in (c) MCH/THF and (d) THF solution at the same condition ($C = 2 \times 10^{-4}$ M, optical path length: 1 mm). All solutions are placed in a cuvette with screw cap and purged with a stream of argon for 30 min before photoreaction.

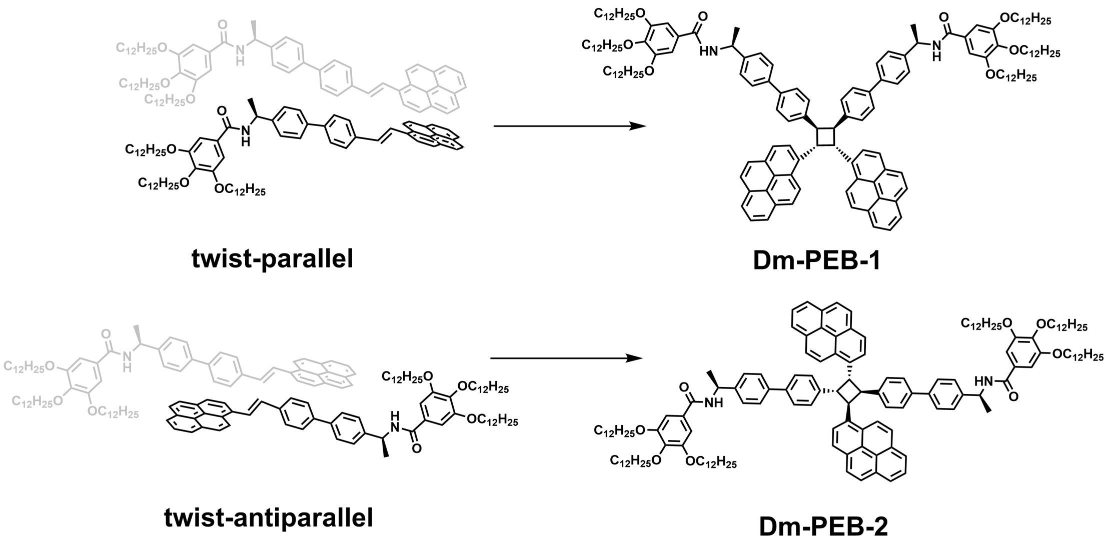

Supplementary Figure 27. Two possible packing modes of *S*-PEB and the corresponding [2+2] cycloaddition products upon light irradiation. Two of the photoproducts (Dm-PEB-1 and Dm-PEB-2) are energetically favored structures. The "twist-antiparallel" arrangement is the major stacking mode, confirmed by the experimental data and theoretical analysis. Dm-PEB-2 is a result of a favored trans-trans cycloaddition. These results are in accordance with the previous literature.[3]

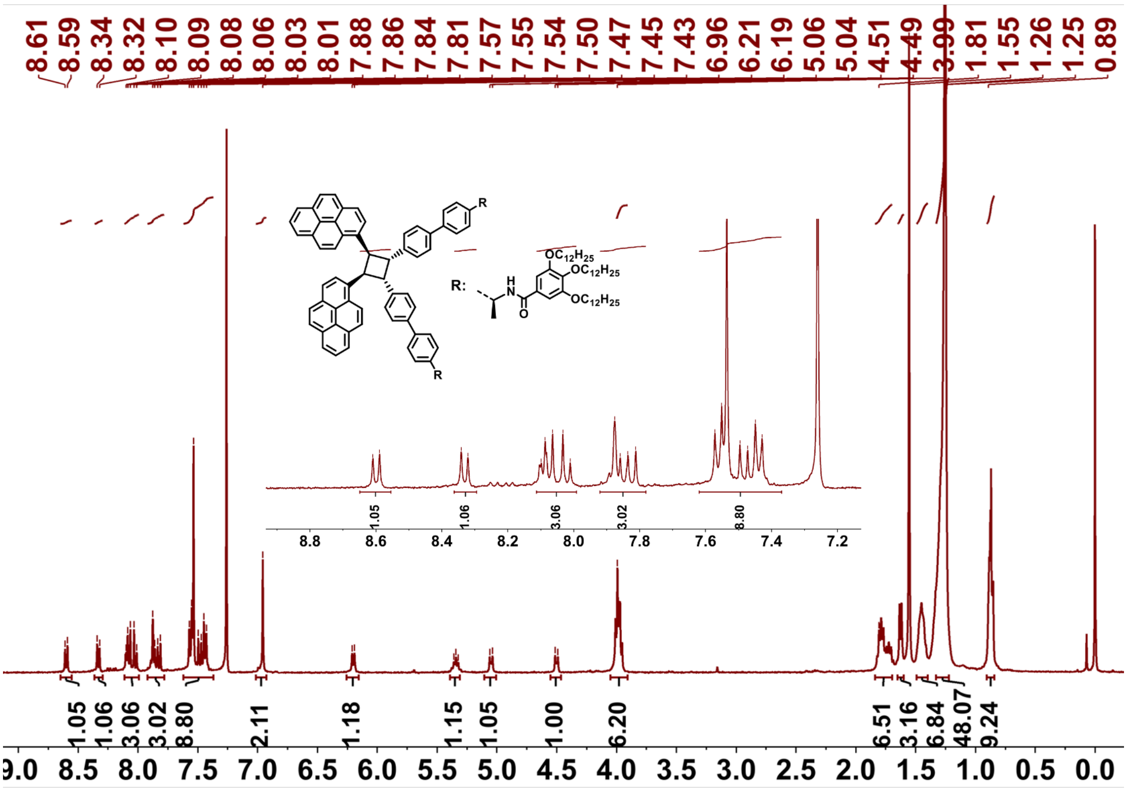

Supplementary Figure 28. ^{1}H NMR spectrum of compound Dm-PEB-1 in $CDCl_3$.

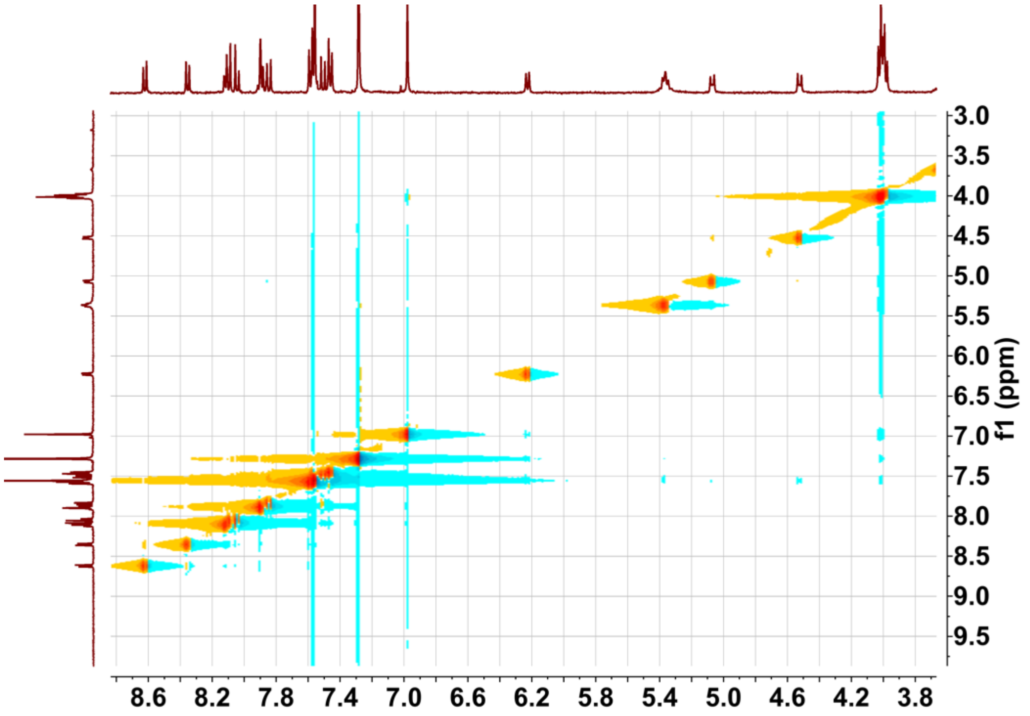

Supplementary Figure 29. NOESY measurement of compound Dm-PEB-1 in $CDCl_3$.

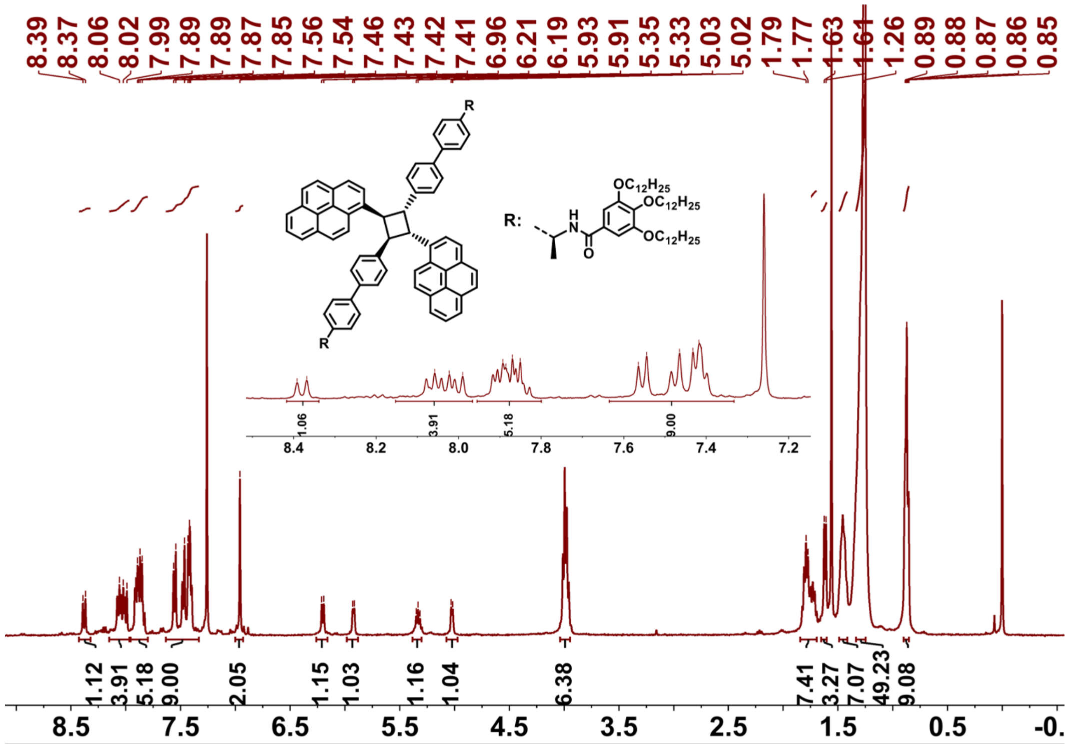

Supplementary Figure 30. ^{1}H NMR spectrum of compound Dm-PEB-2 in $CDCl_3$.

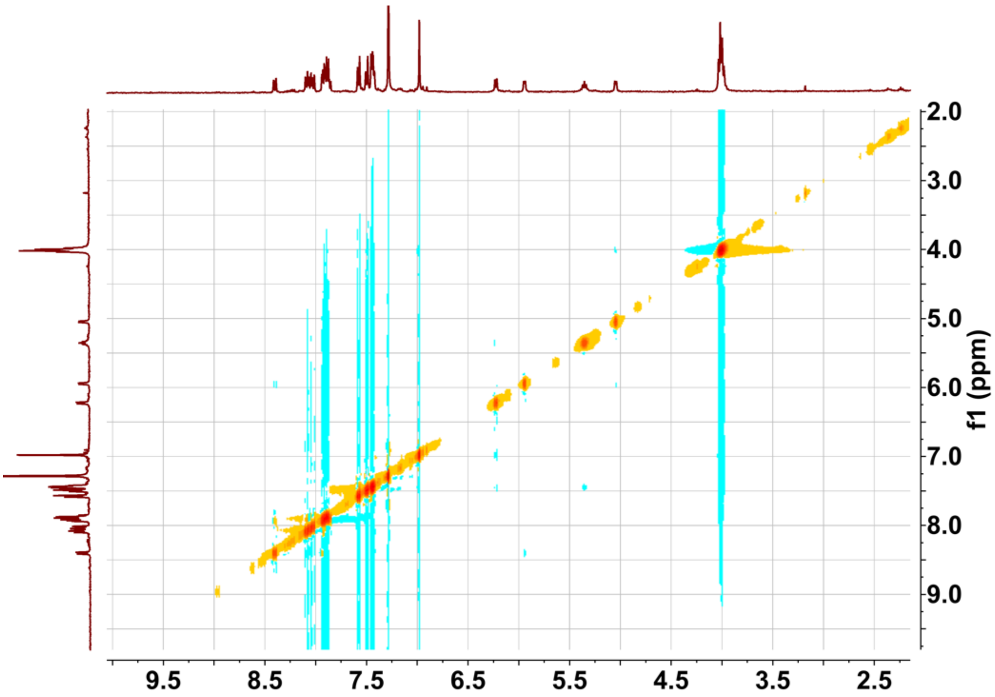

Supplementary Figure 31. NOESY measurement of compound Dm-PEB-2 in $CDCl_3$.

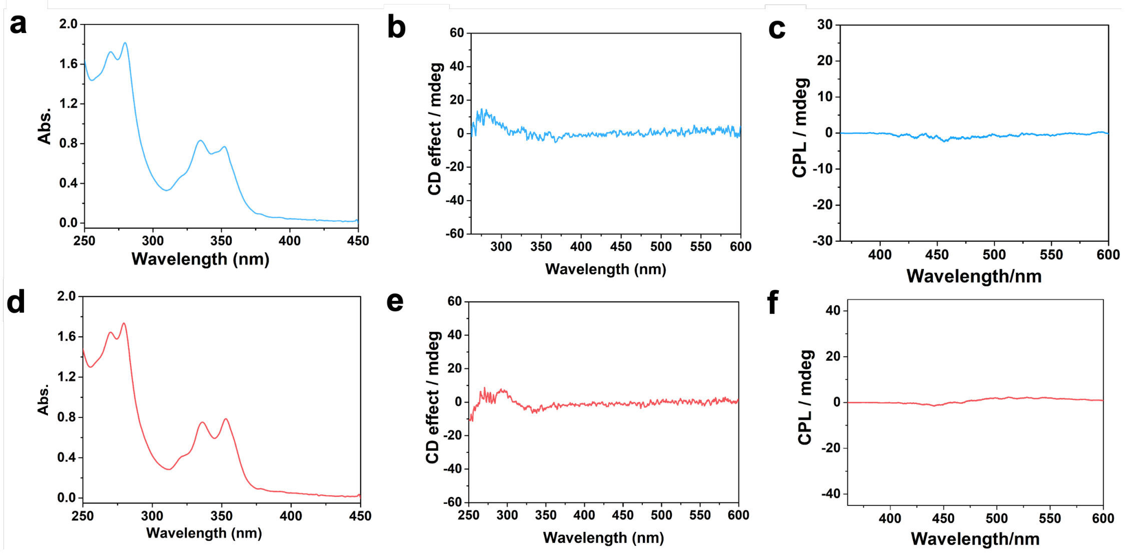

Supplementary Figure 32. **UV-vis, CD and CPL spectra.** UV-vis (a), CD (b) and CPL (c) spectra of Dm-PEB-1 in MCH solution. UV-vis (d), CD (e) and CPL (f) spectra of Dm-PEB-1 in MCH solution. All the concentrations are 2.5×10^{-4} M.

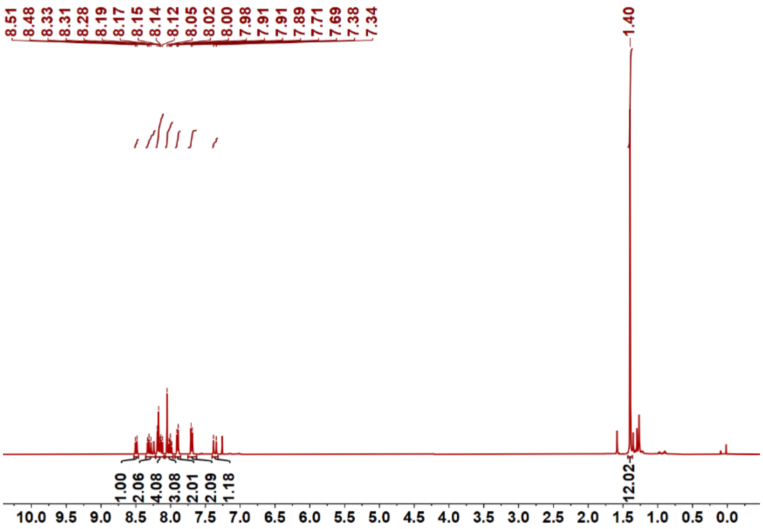

Supplementary Figure 33. ^{1}H NMR spectrum of compound B_1 in $CDCl_3$.

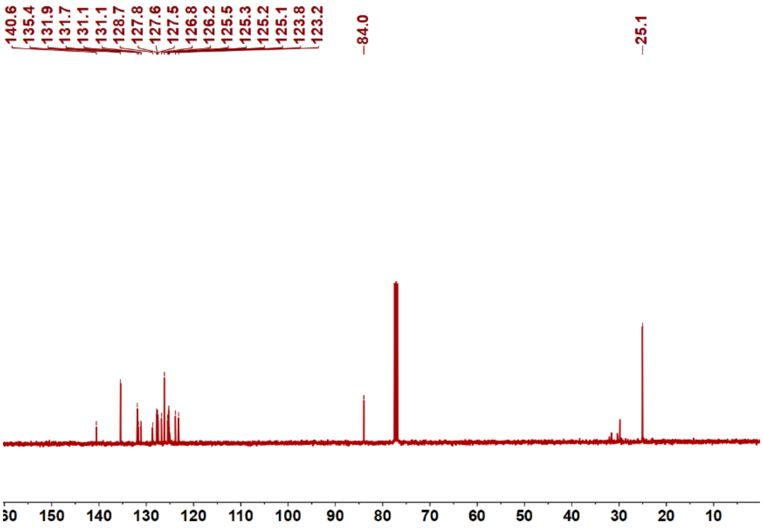

Supplementary Figure 34. ^{13}C NMR spectrum of compound B_1 in $CDCl_3$.

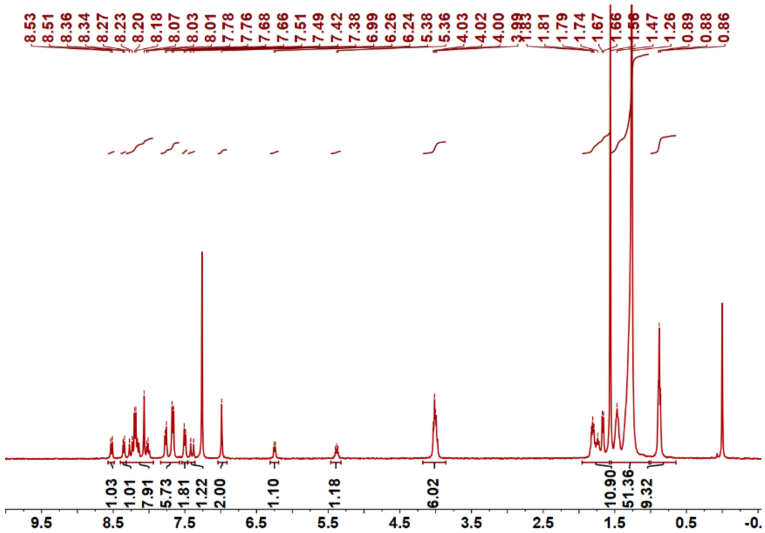

Supplementary Figure 35. ^{1}H NMR spectrum of compound *S*-PEB in $CDCl_3$.

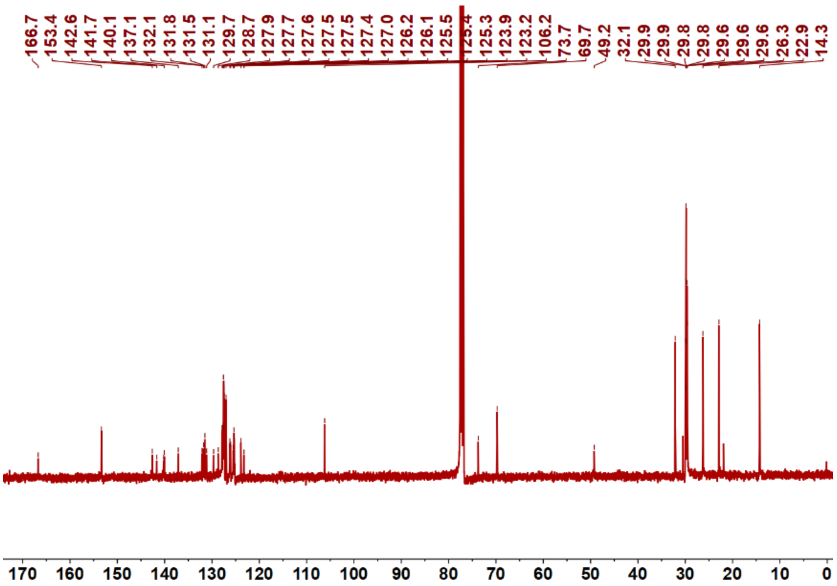

Supplementary Figure 36. ^{13}C NMR spectrum of compound *S*-PEB in $CDCl_3$.

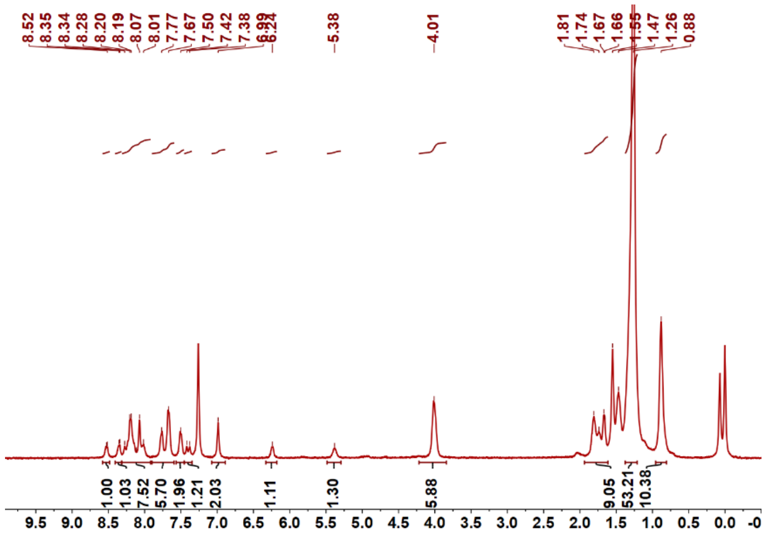

Supplementary Figure 37. ^{1}H NMR spectrum of compound *R*-PEB in $CDCl_3$.

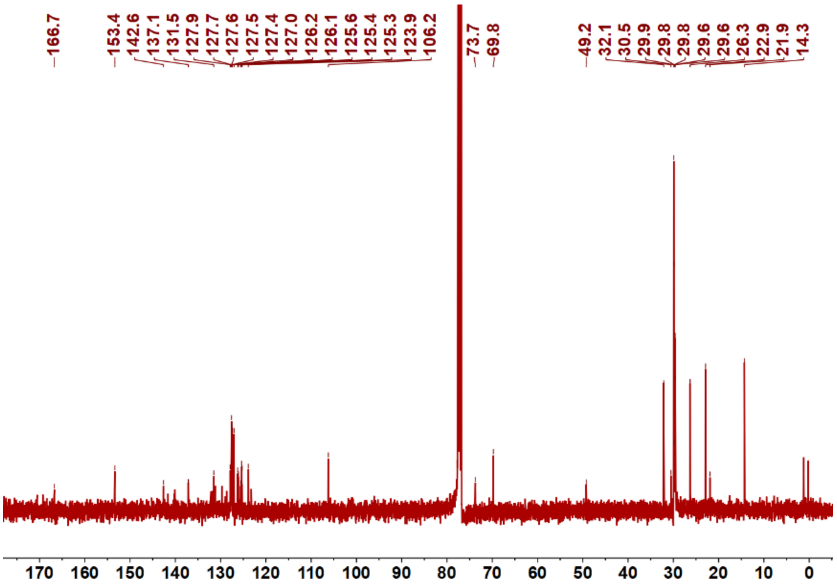

Supplementary Figure 38. ^{13}C NMR spectrum of compound *R*-PEB in $CDCl_3$.

Supplementary References

1. Gong, Z.-L. & Zhong, Y.-W. Handedness-inverted polymorphic helical assembly and circularly polarized luminescence of chiral platinum complexes. *Sci. China Chem.* **64**, 788-799 (2021).
2. Humphrey, W., Dalke, A. & Schulten, K. VMD: Visual molecular dynamics. *J. Mol. Graphics* **14**, 33-38 (1996).
3. Marschner, D.E., Frisch, H., Offenloch, J.T., Tuten, B.T., Becer, C.R., Walther, A., Goldmann, A.S., Tzvetkova, P. & Barner-Kowollik, C. Visible light [2 + 2] cycloadditions for reversible polymer ligation. *Macromolecules* **51**, 3802-3807 (2018).
